# Comparison and evaluation of neutralization of clinically frequently used antimicrobial agents using three different culture media in simulated blood cultures

**DOI:** 10.1128/spectrum.00979-24

**Published:** 2024-08-27

**Authors:** Yaping Hang, Jianqiu Xiong, Longhua Hu, Yanhui Chen, Shan Zou, Xueyao Fang, Yanping Xiao, Xingwei Cao, Hong Lou, Xiuzhen Li, Yanhua Liu, Qiaoshi Zhong

**Affiliations:** 1 Jiangxi Province Key Laboratory of Immunology and Inflammation, Jiangxi Provincial Clinical Research Center for Laboratory Medicine, Department of Clinical Laboratory, The 2nd Affiliated Hospital, Jiangxi Medical College, Nanchang University, Nanchang, China; 2 Intravenous Medication Dispensing Center, The 2nd Affiliated Hospital, Jiangxi Medical College, Nanchang University, Nanchang, China; NHLS Tygerberg/Stellenbosch University, Cape Town, Western Cape, South Africa

**Keywords:** BACT/ALERT plus media, DL media, REDOX media, blood culture, antimicrobial neutralization, recovery rate, aerobic, anaerobic

## Abstract

The performance of BACT/ALERT FA/FN Plus (France) blood culture containing a novel resin, DL (China) blood culture containing common resin, and adsorbent-free REDOX (USA) blood culture relying on dilution for antimicrobial neutralization at %peak serum concentration was evaluated by measuring the recovery of organisms and time to detection (TTD) in nine simulated microorganism-antimicrobial combination blood cultures. Significant differences were observed in the recovery rates among the aerobic media: 87.5% for BACT/ALERT media, 42.9% for DL media, and 12.5% for REDOX media. In contrast, no statistical difference was found in the TTD between FA Plus media and DL aerobic media. For the anaerobic media, the recovery rates were 91.4% for BACT/ALERT media, 2.9% for DL media, and 14.3% for REDOX media, with significant differences only between BACT/ALERT FN Plus media and the others. Among the seven main antimicrobial categories, only BACT/ALERT FA/FN Plus culture media demonstrated high recovery of microorganisms, with the exception of carbapenems. The DL culture media exhibited a relatively high recovery rate of microorganisms in the presence of piperacillin/tazobactam, levofloxacin, and gentamicin, but only in aerobic conditions. Conversely, REDOX media displayed microorganism recovery solely in the presence of gentamicin. BACT/ALERT FA/FN Plus culture media with novel resin showed absolute advantages over DL and REDOX culture media and can, therefore, be selectively applied in clinical settings when antimicrobials are used prior to blood collection. DL culture media, containing common resin, outperformed adsorbent-free dilution-based REDOX culture media, making it a viable backup option. There is a need to focus on improving the neutralization of carbapenems with current inefficiency in all three medias.

**IMPORTANCE:**

We present a study on performance comparison of three different commercial culture media for neutralization of antibiotic effects in simulated blood cultures. BACT/ALERT (FA Plus and FN Plus) culture media with novel resin showed absolute advantages over DL and REDOX culture media at %PSL concentration of antimicrobials.

## INTRODUCTION

Accurate and timely diagnosis of bloodstream infections (BSIs) is crucial for the proper management of patients, particularly those with sepsis and septic shock complications, and for reducing the associated high morbidity and mortality (1). Blood cultures remain the gold standard for BSIs detection and should be collected before administering antimicrobial drugs when sepsis is suspected in patients. However, in urgent cases of sepsis, the supervising physician may provide broad-spectrum antimicrobial therapy empirically before obtaining a microbiological diagnosis of the infection (2). To minimize the inhibitory effects of antimicrobials on microbial growth in blood cultures, certain commercial blood culture media have been developed that contain substances, such as resin or charcoal. These substances are intended to adsorb antimicrobials or other inhibitory substances, thereby improving the detection of microorganisms in samples from sepsis patients (3, 4). While most studies primarily focused on the capacity of bacterial pathogenic detection in blood cultures, some recent simulation studies have assessed the antimicrobial-neutralizing capabilities of various blood culture media. These studies involved media injected with whole blood, antimicrobials, and microorganisms, and they evaluated different mechanisms used by various manufacturers (5, 6).

Numerous techniques have been developed to neutralize antimicrobials in blood culture media, each with its own set of disadvantages (3, 4). In contrast to the single adsorption principle of activated charcoal and common resins, culture media with APB resin and ZL covalent bond additives can effectively meet the neutralization requirements for antimicrobials without disrupting the trace elements and nutrients essential for bacterial growth (7–12). The BACT/ALERT media (FA Plus and FN Plus) contain the enhanced ZLAPB resin for antimicrobial adsorption. The adsorption of various antimicrobials depends on three distinct pathways: synergistic ion exchange, van der Waals forces, and covalent bonds. These pathways optimize the adsorption performance for a range of antimicrobials, including cephalosporins, imipenem, meropenem, daptomycin, cephalosporin, carbendazim, and other high-order antimicrobial agents. The antimicrobial neutralization of DL media relies on common resins, which can also adsorb some of the antimicrobials. However, the overall performance of the REDOX media without any adsorbent depends on an optimal 1:9 blood-broth dilution. As a result, antimicrobial neutralization capacity is limited.

This study aimed to compare the neutralization effects of three automated blood culture systems that utilize three manufacturer blood culture media with different antimicrobial neutralization mechanisms: including approved China mainland BACT/ALERT (FA Plus and FN Plus) media (bioMérieux, France) since 2019, DL widely used in China mainland (ZhuhaiDL, China), and REDOX blood culture media (Thermo Scientific, USA).

## MATERIALS AND METHODS

### Blood culture media and instruments

In this study, all the inoculated BACT/ALERT blood culture media including FA Plus aerobic, FN Plus anaerobic, SA aerobic (SA), and SN anaerobic (SN) were applied to the automated BACT/ALERT 3D blood culture system. DL blood culture media (commonly used in China) was utilized with an automated DL-Bt blood culture system. Thermo VersaTREK REDOX 1 Aerobic (REDOX 1) and Thermo VersaTREK REDOX 2 Anaerobic (REDOX 2) blood culture media were evaluated using an automated Thermo Scientific VersaTREK blood culture system. Among all the blood culture media, the volume of BACT/ALERT medium is 40 mL and the volume of DL medium is 30 mL, whereas the volume of REDOX medium is 80 mL. In the term of antimicrobial neutralization, BACT/ALERT FA/FN Plus media contain ZLAPB resin adsorbent, while DL media contain common resin adsorbent; however, BACT/ALERT SA/SN medium and REDOX medium are free of any adsorbent.

### Microorganisms and antimicrobial substances

Microbial species commonly isolated in clinical microbiology laboratories were selected for this study. The reference strains included methicillin-susceptible *Staphylococcus aureus* ATCC 29213, *Streptococcus pneumoniae* ATCC 49619, *Escherichia coli* ATCC 25922, *Pseudomonas aeruginosa* ATCC 27853, *Candida albicans* ATCC 90028, and *Bacteroides fragilis* ATCC 25285. The susceptibility of corresponding ATCC strains to the antimicrobial agents used in this study was tested. Colonies from Columbia blood agar plates were suspended in physiological saline and serially diluted to achieve a target suspension of 10^2^ CFU/mL (CFU,colony-forming unit). Concurrently, 0.3 mL of the final dilutions were plated on Columbia blood agar plates and incubated at 37°C overnight to validate the CFUs. All the target diluted suspension were satisfied the inoculation requirement at 30-100 CFU/bottle, and the inoculation concentration was in accordance with the Antibiotic Neutralisation Capacity Assessment Programme.

The nine antimicrobials most commonly used to treat specific BSIs were selected, as listed in Table 1. Standards of penicillin G, piperacillin/tazobactam, meropenem, imipenem, gentamicin, cefoxitin, levofloxacin, vancomycin, and fluconazole (Meilunbio, China) were dissolved in appropriate solvents according to Clinical and Laboratory Standards Institute (CLSI) guidelines and the manufacturer’s instructions. Each antimicrobial agent was diluted with sterile water or phosphate-buffered saline (PBS) from stock solutions to ensure that 0.5 mL of the dilution achieved the desired final drug concentration for each culture media. Antimicrobial solutions at peak serum concentrations (%PSL) following standard adult dosing were utilized to simulate patient blood levels, in accordance with CLSI M100, M60, CNAS-GL028, and the Sanford Guide to Antimicrobial Therapy, 50th Edition (Table 1).

**TABLE 1:** Microorganism and antimicrobial combination tested by the simulated adult blood culture model

Species (drug)	Antibiotic	Species (strain)	Atmosphere		PBS/blood	MIC^*a*^ (μg/mL)	PSL^*b*^ (μg/mL)	%PSL^*c*^
			Aerobic	Anaerobic				
Penicillins	Penicillin G	*Streptococcus pneumoniae* (ATCC 49619)	√	√	Blood	0.25–1 (S)	20	100
	Piperacillin/tazobactam	*Pseudomonas aeruginosa* (ATCC 27853)	√		PBS	1/4–8/4 (S)	242/24	100
Carbapenems	Meropenem	*Bacteroides fragilis* (ATCC 25285)		√	Blood	0.03–0.25 (S)	49	100
	Imipenem	*Escherichia coli* (ATCC 25922)	√	√	PBS	0.06–0.25 (S)	40	100
Aminoglycosides	Gentamicin	*Escherichia coli* (ATCC 25922)	√	√	PBS	0.25–1 (S)	10	100
Cephamycins	Cefoxitin	*Staphylococcus aureus* (ATCC 29213)	√	√	PBS	1–4 (S)	110	100
Quinolones	Levofloxacin	*Staphylococcus aureus* (ATCC 29213)	√	√	PBS	0.06–0.5 (S)	8.6	100
Glycopeptides	Vancomycin	*Staphylococcus aureus* (ATCC 29213)	√	√	PBS	0.5–2 (S)	50	100
Triazoles	Fluconazole	*Candida albicans* (ATCC 90028)	√		PBS	0.25–1 (S)	14	100

^*a*^ Minimum Inhibitory Concentrations (MICs) of the antimicrobial drugs for each strain tested were determined by CLSI broth microdilution and susceptibility categorization was assigned based on published CLSI breakpoints. S, susceptible.

^*b*^ PSL, peak serum concentration.

^*c*^ %PSL, drug effective concentration based by the plasma protein binding ability and excretion rates of drugs.

### Blood culture

In this study, blood culture media were inoculated with 9 mL of PBS or sterile horse blood (Oxoid), 0.3 mL of microorganism suspension (10^2^ CFU/mL), and 0.5 mL of antimicrobial solution at %PSL. Microorganisms were simultaneously spiked into both aerobic and anaerobic media as pairs, except for *P. aeruginosa* and *C. albicans*, which were only evaluated under aerobic conditions, and *B. fragilis,* which was tested under anaerobic conditions. *Candida.* spp. exhibited poor growth in anaerobic media, due to their nature as facultative anaerobes with extended generation times under such conditions, which could affect the recovery of numerous pathogens, especially when exposed to antimicrobial agents (13). Consequently, the recovery of *C. albicans* in the presence of fluconazole was excluded from anaerobic media.

For each microorganism-antimicrobial combination, five assays for each combination were realized to assess the antimicrobial neutralization in three different blood culture medias, and triplicate for each contrast test. The contrast tests included positive contrast tests, which involved incubating bacterial suspension in three different blood culture medias; antimicrobial efficacy contrast tests, to confirm the efficacy and susceptibility of the tested antimicrobials, performed by incubating microorganism-antimicrobial combinations in adsorbent-free SA/SN standard media; and negative antimicrobial-free contrast tests, conducted by incubating only PBS or horse blood in SA/SN bottles.

All inoculated blood culture media were incubated in corresponding blood culture system within 5 days; the blood culture medias were inoculated in blood columbia blood agar plates overnight after they were flagged as either positive, then microorganisms from positive blood culture were then identified by their morphological characteristics and with DL mass spectrometry to exclude contamination; Subsequently, broth from negative blood culture bottles was subcultured onto Columbia blood agar plates overnight to confirm the absence or presence of growth.

### Data analysis and statistics

Statistical analyses were performed using GraphPad Prism 7. The detection rates in aerobic and anaerobic media between the three culture systems were compared using Fisher’s exact test. Mann-Whitney U-tests were applied to analyze variations in time to detection (TTD), and the median of TTD was compared due to the non-normal distribution of the data. A *P-*value of <0.05 was considered to indicate statistical significance.

## RESULTS

**Fig 1:**
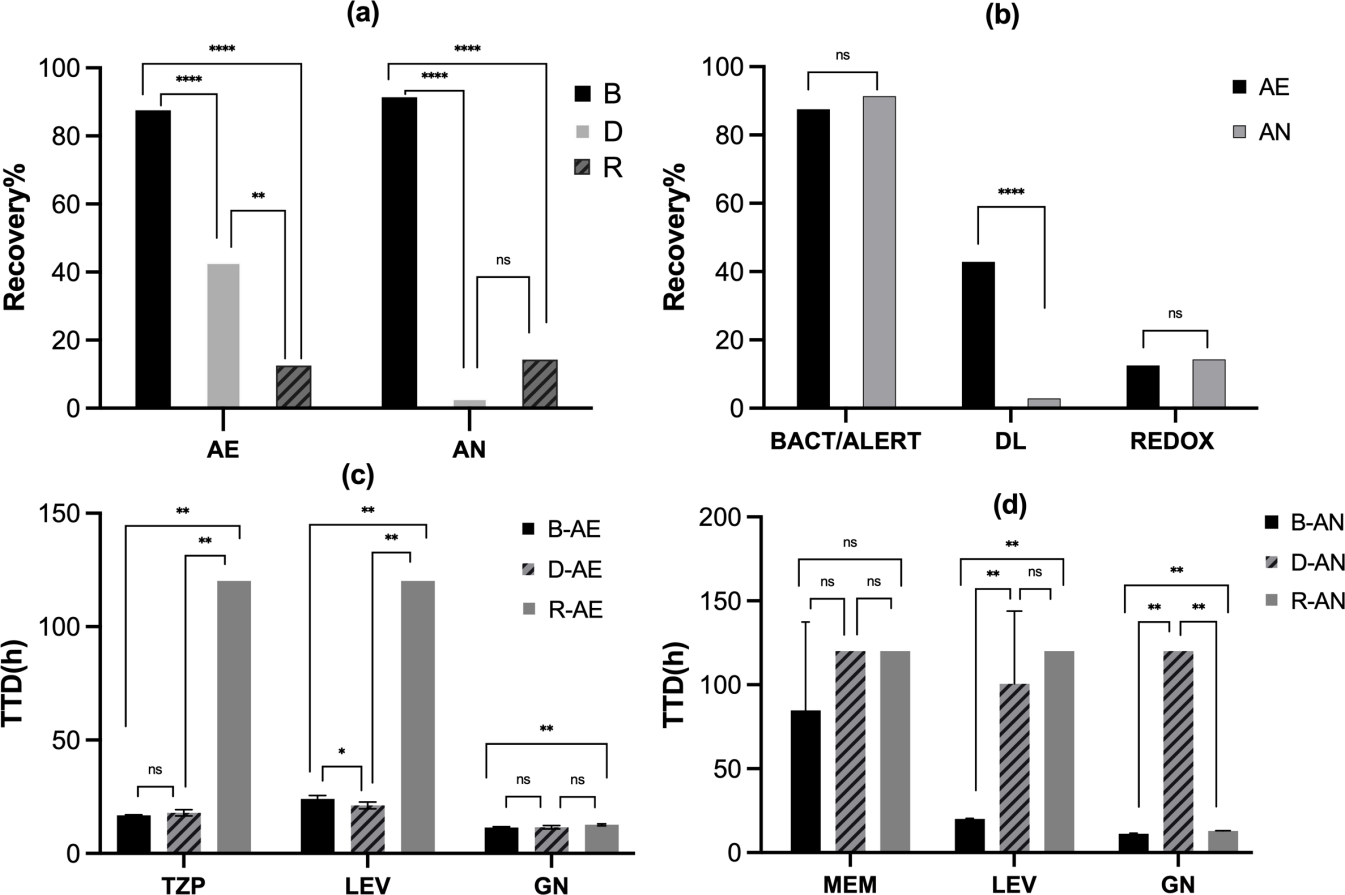
(a) Recovery rate of microorganisms in aerobic (BACT/ALERT Plus, DL, and REDOX) or anaerobic (BACT/ALERT Plus, DL, and REDOX) culture media containing antimicrobials. (b) Recovery rate of microorganisms in different (aerobic and anaerobic) culture media containing antimicrobials. (c) TTD of microorganisms recovered from BACT/ALERT FA Plus, DL aerobic, and REDOX 1 media containing different antimicrobials [piperacillin/tazobactam (TZP), levofloxacin (LEV), and gentamicin (GN)]. (d) TTD of microorganisms recovered from BACT/ALERT FN Plus, DL anaerobic, and REDOX 2 media containing different antimicrobials [meropenem (MEM), LEV, and GN]. Abbreviations: B, BACT/ALERT culture (FA Plus and FN Plus) media; D, DL culture media; R, REDOX culture media; AE, aerobic media; AN, anaerobic media; ns, not significant; *, *P*  <  0.05; **, *P* < 0.01; ***, *P* < 0.001; ****, *P* <  0.0001. Error bars indicate standard deviations (SD).

In the BACT/ALERT 3D blood culture system, 40 FA Plus media and 35 FN Plus media were tested. In the DL-Bt blood culture system, 35 DL aerobic and 35 DL anaerobic media were examined, and in the Thermo Scientific VersaTREK blood culture system, 40 REDOX 1 and 35 REDOX 2 media were evaluated. All microorganisms tested in the antimicrobial-free control media were recovered, except for *C. albicans* in the DL aerobic media, which had a recovery rate of 97.8%. Furthermore, the positive controls of recovering *C. albicans* in DL aerobic media were negative. Consequently, the *C. albicans*-fluconazole combination in DL media was excluded. When antibiotics were present, eight of the nine antimicrobial agents exhibited adequate antibacterial activity, and all showed no microorganism growth except for gentamicin in adsorbent-free BACT/ALERT SA media. The overall recovery rate of FA Plus media was 87.5%, which was significantly higher than that of DL aerobic media (42.9%; *P* < 0.001) and REDOX 1 media (12.5%; *P* < 0.001). In anaerobic cultures, the overall recovery rate of FN Plus media was 97.4%, which was higher than that of 2.9% of DL anaerobic media (*P* < 0.001) and 14.3% of REDOX 2 media (*P* < 0.001; Table 2; Fig. 1a). The neutralizing effects of the antimicrobial agents in both aerobic and anaerobic media from the same manufacturer were consistent, except that the recovery rate of the DL aerobic media was higher than that of the anaerobic media (42.9% vs 2.9%, *P* < 0.001; Table 2; Fig. 1b).

**TABLE 2:** Time to detection of microorganisms recovered from three different aerobic and anaerobic media at %PSL concentration tested in blood culture quintuplicates^*f*^

ATCC strain/antimicrobial	BACT/ALERT Plus media						DL media						REDOX media					
	Recovery^*a*^		Median TTD^*b*^ (h)		∆TTD^*c*^ (h)		Recovery^*a*^		Median TTD^*b*^ (h)		∆TTD^*c*^ (h)		Recovery^*a*^		Median TTD^*b*^ (h)		∆TTD^*c*^ (h)	
	AE	AN	AE	AN	AE	AN	AE	AN	AE	AN	AE	AN	AE	AN	AE	AN	AE	AN
*S. pneumoniae*																		
Penicillin G	5/5	5/5	15.12	16.08	1.20	1.92	0/5	0/5	>120	>120	NA^*d*^	NA	0/5	0/5	>120	>120	NA	NA
*P. aeruginosa*																		
Piperacillin/tazobactam	5/5	− ^*e*^	16.80	−	0.48	−	5/5	−	18.35	−	1.12	−	0/5	−	>120	−	NA	−
*B. fragilis*																		
Meropenem	−	2/5	−	31.92	−	−7.44	−	0/5	−	>120	−	NA	−	0/5	−	>120	−	NA
*E. coli*																		
Imipenem	0/5	5/5	>120	10.8	NA	−0.48	0/5	0/5	>120	>120	NA	NA	0/5	0/5	>120	>120	NA	NA
Gentamicin	5/5	5/5	11.52	11.28	0.24	−0.24	5/5	0/5	11.25	>120	0.86	NA	5/5	5/5	12.70	12.70	1.2	−0.4
*S. aureus*																		
Cefoxitin	5/5	5/5	41.5	29.52	19.92	9.12	0/5	0/5	>120	>120	NA	NA	0/5	0/5	>120	>120	NA	NA
Levofloxacin	5/5	5/5	23.52	20.16	1.44	−0.72	5/5	1/5	20.57	23.00	7.84	10.77	0/5	0/5	>120	>120	NA	NA
Vancomycin	5/5	5/5	23.52	20.40	0.00	0.48	0/5	0/5	>120	>120	NA	NA	0/5	0/5	>120	>120	NA	NA
*C. albicans*																		
Fluconazole	5/5	−	17.52	−	−2.4	−	−	−	−	−	−	−	0/5	−	>120	−	NA	−

^*a*^ No. of replicates/total.

^*b*^ For each microorganism-antimicrobial combination tested, the median TTD was calculated by summing the TTDs of single blood culture replicates. TTD values are rounded to the nearest decimal point.

^*c*^ For each microorganism-antimicrobial combination tested, ΔTTD is the difference in TTD between the microorganism-antimicrobial-containing blood culture and the antimicrobial-free blood culture (antimicrobial-free control).

^*d*^ NA, not applicable (no microorganism growth in any bottle).

^*e*^ Lack of data because the microorganisms were cultured only in aerobic or anaerobic media.

^*f*^ AE, aerobic media; AN, anaerobic media.

Medias inoculated with gentamicin demonstrated the highest recovery rate, as nearly all the tested strains were recovered in both culture media except for the DL anaerobic bottle. The BACT/ALERT (FA Plus and FN Plus) culture media showed a slightly shorter TTD of gentamicin than REDOX culture media (*P* = 0.008 ; Table 2; Fig. 1c and d). Medias inoculated with piperacillin/tazobactam and levofloxacin achieved the highest recovery rate, with all the test strains being recovered in BACT/ALERT Plus media and DL media; TTD of medias inoculated with piperacillin/tazobactam in aerobic atmosphere showed no significant difference between the two culture media; BACT/ALERT FN Plus culture showed slightly shorter TTD (−2.8 h) of levofloxacin than DL anaerobic culture media (*P* = 0.008), whereas BACT/ALERT FA Plus culture showed slightly longer TTD (3.0 h) of levofloxacin than DL aerobic culture media (*P* < 0.008; Table 2; Fig. 1c and d). Moreover, only the BACT/ALERT system demonstrated 100% recovery with nearly all the tested antimicrobial agents, except carbapenems, which showed significantly higher recovery rates than the DL system (penicillin G, *P* < 0.001; cefoxitin, *P* < 0.001; vancomycin, *P* < 0.001; Table 2). For carbapenems, medias inoculated with meropenem exhibited the lowest recovery rate in all blood cultures (13.3%), and most of the microorganisms were only recovered from BACT/ALERT FN Plus media (40.0% vs 0.0% vs 0.0%, *P* = 0.44). A similar pattern was observed for medias inoculated with imipenem, which had low recovery rate in all blood cultures (16.7%), and microorganisms were only recovered from BACT/ALERT FN Plus media (50.0% vs. 0.0% vs. 0.0%, *P* = 0.033). For fluconazole, microorganisms were exclusively recovered from BACT/ALERT FN Plus media.

Our research also focused on comparing TTD between BACT/ALERT Plus media and DL media, as REDOX media only exhibited the recovery of organisms with gentamicin. Organisms were still recovered in the presence of antimicrobials for seven microorganism-antimicrobial combinations in both aerobic culture systems. Moreover, shorter TTD was observed in FA Plus culture media compared to DL aerobic culture media for four (57.1%) of these combinations. Between the two anaerobic culture systems, organism recovery was possible in seven microorganism-antimicrobial combinations, with all seven indicating shorter TTD in FN Plus media compared with DL anaerobic media (Table 2). Additionally, no statistically significant difference in TTD was observed between FA Plus and DL aerobic media (*P* = 0.350).

Table 2 summarizes the differences in TTDs between media spiked with microorganism-antimicrobial combinations and antimicrobial-free media (ΔTTD) (14). For BACT/ALERT (FA Plus and FN Plus) media, the highest ΔTTD (19.9 h aerobic, 9.1 h anaerobic) was detected for cefoxitin-*S. aureu*s, whereas for DL media, the ΔTTD of levofloxacin-*S. aureus* was also notable (7.8 h aerobic, 10.8 h anaerobic). Negative ΔTTDs were observed in certain microorganism-antimicrobial combinations, particularly fluconazole-*C. albicans* (−2.4 h) in BACT/ALERT FA Plus media and meropenem-*B. fragilis* (−7.4 h) in BACT/ALERT FN Plus media.

## DISCUSSION

We observed significantly higher microorganism recovery rates with the BACT/ALERT Plus media than the other two culture media (DL and REDOX) that contained antimicrobials, in both aerobic and anaerobic cultures. Similarly, there was a higher detection rate for microorganisms in at least one bottle of each pair. The BACT/ALERT system exhibited a more satisfactory detection rate and higher recovery rates than those reported in other studies (5, 6), which was probably due to the larger inoculum (30–10^2^ CFU/bottle), the appropriate inoculation volume (approximately 10 mL), and the use of susceptible ATCC strains in our research. Almost all antibacterial categories were successfully neutralized in both BACT/ALERT (FA Plus and FN Plus) culture media, with the exception of carbapenems. For carbapenems, the recovery rate of microorganisms was lowest and only recovered in BACT/ALERT FN Plus media. This outcome was expected to be affected by carbapenem neutralization of BACT/ALERT FN Plus media, which relies on the adsorption through ZL cysteine covalent bonds; cysteine is essential for anaerobic environments (7–9, 14, 15). However, this specific resin is not to be present in other medias. In line with previous studies on BACT/ALERT (FA Plus and FN Plus) culture media, the detection rates of microbial-imipenem and microbial-meropenem detection rates were lower at 5/10 (50.0%) and 2/5 (40%), respectively (5, 16). Our result indicated the lowest recovery rate of *B. fragilis* with meropenem in BACT/ALERT blood culture. A potential explanation for this is the low MIC and high Cmax/MIC quotient for the strain-antimicrobial combination (Table 1). These data suggest that the variability in performances across the studies may depend not only on antimicrobial-binding media but also on the specific combinations of bacteria and antibiotics (17).

We also observed that the levofloxacin in free %PSL concentration quickly fell below the strain’s MIC range in BACT/ALERT FA Plus media within 10 min of incubation (16). Therefore, to prevent the majority of antimicrobials from being absorbed, a bacterial suspension was added before the antimicrobial solution. This approach resulted in a more consistent recovery rate of the *S. aureus-*levofloxacin combination in BACT/ALERT (FA Plus and FN Plus) culture media compared to previous studies (6, 18).

For the domestically manufactured DL culture media with common resin, we further excluded the recovery of *C. albicans* in the presence of fluconazole in aerobic media as the positive controls failed, indicating that *C. albicans* cannot be recovered from DL culture media free of fluconazole.The DL aerobic culture media with common resin demonstrated better and higher recovery rates in the presence of piperacillin/tazobactam and levofloxacin, in comparison with DL anaerobic and REDOX media. A significant difference (*P* < 0.001) in antimicrobial neutralization was observed between the DL aerobic and anaerobic media due to the variation in the oxygen consumption niche of spiked microorganisms.

The overall performance of the REDOX media without any adsorbent was inferior compared to the other two media owing to its lower neutralizing activity. It depends on an optimal 1:9 blood-broth dilution for neutralizing antimicrobial agents. However, the observations indicated a high recovery rate of the microorganism-gentamicin combination in all three media. This is likely because gentamicin is easily affected by factors such as oxygen consumption, Sodium polyanisaldehyde sulfonate (SPS) anticoagulant, antimicrobial adsorbent, and dilution.

TTD is a significant parameter to assess the performance of commercial automatic blood culture systems (5, 6, 19). Generally, BACT/ALERT FN Plus media demonstrated shorter TTDs than DL anaerobic media because of higher microbial recovery rates. Moreover, there was no statistically significant difference in TTDs between FA Plus media and DL aerobic media. The BACT/ALERT FA Plus media did not outperform DL aerobic media in TTDs, given that their microbial recovery rates were nearly equivalent. For gentamicin, BACT/ALERT (FA plus and FN Plus) culture media showed a slightly shorter TTD than the REDOX media. Nonetheless, the potential impact of the typical several-hour differences on decision-making in clinical settings remains uncertain. Further prospective clinical research is required to evaluate the influence of TTD on the initiating or modifying antibiotic treatment strategies.

Regarding ΔTTD, another crucial parameter to assess the antimicrobial inactivation ability of the examined automatic blood culture media (14), delays of at least 3 h compared to positive results in control (antimicrobial-free) blood culture media were observed only for cefoxitin-*S. aureu*s in the BACT/ALERT (FA Plus and FN Plus) culture media pair and for levofloxacin-*S. aureus* in the DL culture media pair. Negative ΔTTDs were also noted in certain microorganism-antimicrobial combinations, specifically in fluconazole-*C. albicans* (−2.4 h) in BACT/ALERT FA Plus media, consistent with previous reports (6). Moreover, negative ΔTTDs for meropenem-*B. fragilis* (−7.4 h) in BACT/ALERT and FN Plus media were likely influenced by the novel resin ZLAPB. Furthermore, delayed ΔTTDs were more frequently observed in aerobic media. Therefore, the efficacy of antimicrobial inactivation may be affected by the type of antimicrobials and/or microbial species, as well as the interaction within the complex environment of blood culture media.

This study has certain limitations. Firstly, horse blood was utilized for the simulation instead of human blood. Secondly, only a limited number of isolates were tested, and the evaluation focused solely on susceptible ATCC strains and did not include clinical isolates. Only a restricted number of bacterial and antimicrobial combinations were assessed, and those frequently isolated in a clinical setting, including methicillin-resistant *S. aureus* MRSA, enterococci, and filamentous fungi, were not examined. Lastly, the neutralization of three blood culture media was assessed at solely %PSL concentration of antimicrobials. Further studies encompassing a broader range of microbial and varying concentrations of antimicrobials are necessary to provide more comprehensive insights into the comparative evaluation of blood culture media for antimicrobial neutralization.

### Conclusion

Absolute advantages were revealed in our simulated study using BACT/ALERT (FA Plus and FN Plus) media containing the novel resin at %PSL concentration of antimicrobials. With the exception of carbapenems, most of the tested broad-spectrum antimicrobial agents at clinically relevant concentrations were effectively neutralized. To improve the chances of recovery for patients who have previously received antimicrobial therapy, relatively more costly media (FA Plus and FN Plus) could be selectively utilized. The results indicated that the DL aerobic culture medium with common resin was significantly more efficient in recovering challenge organisms with piperacillin/tazobactam and levofloxacin than the adsorbent-free REDOX culture media. Emphasis should be placed on enhancing the neutralization of carbapenems with low efficiency in all three media. Further comparative studies using blood samples from patients undergoing antimicrobial therapy will provide more information on the performance of culture systems in antimicrobial neutralization.
